# Asymmetric effects of grazing intensity on macroelements and microelements in grassland soil and plants in Inner Mongolia Grazing alters nutrient dynamics of grasslands

**DOI:** 10.1002/ece3.6591

**Published:** 2020-07-18

**Authors:** Dongjie Hou, Ke Guo, Changcheng Liu

**Affiliations:** ^1^ State Key Laboratory of Vegetation and Environmental Change, Institute of Botany Chinese Academy of Sciences Beijing China; ^2^ University of Chinese Academy of Sciences Beijing China

**Keywords:** ecological stoichiometry, grazing intensity, macroelement, microelement, typical grassland

## Abstract

Grazing is a traditional grassland management technique and greatly alters ecosystem nutrient cycling. The effects of grazing intensity on the nutrient dynamics of soil and plants in grassland ecosystems remain uncertain, especially among microelements. A 2‐year field grazing experiment was conducted in a typical grassland with four grazing intensities (ungrazed control, light, moderate, and heavy grazing) in Inner Mongolia, China. Nutrient concentration was assessed in soil and three dominant plant species (*Stipa krylovii*, *Leymus chinensis*, and *Cleistogenes squarrosa*). Assessed quantities included four macroelements (carbon (C), nitrogen (N), phosphorus (P), and magnesium (Mg)) and four microelements (copper (Cu), iron (Fe), manganese (Mn), and zinc (Zn)). Soil total C, total N, total P, available N, and available P concentrations significantly increased with grazing intensity but soil Mg, Cu, Fe, Mn, Zn concentrations had no significant response. Plant C concentration decreased but plant N, P, Mg, Cu, Fe, Mn, and Zn concentrations significantly increased with grazing intensity. In soil, macroelement dynamics (i.e., C, N, and P) exhibited higher sensitivity with grazing intensity, conversely in plants, microelements were more sensitive. This result indicates macroelements and microelements in soil and plants had asymmetric responses with grazing intensity. The slopes of nutrient linear regression in *C. squarrosa* were higher than that of *S. krylovii* and *L. chinensis*, indicating that *C. squarrosa* had higher nutrient acquisition capacity and responded more rapidly to heavy grazing. These findings indicate that short‐term heavy grazing accelerates nutrient cycling of the soil–plant system in grassland ecosystems, elucidate the multiple nutrient dynamics of soil and plants with grazing intensity, and emphasize the important function of microelements in plant adaptation in grazing management.

## Graphical abstract

The nutrient dynamics of macroelements and microelements in plants and soil displayed opposite responses to grazing. The slopes of nutrient linear regression in *Cleistogenes squarrosa* were higher than that of *Stipa* krylovii or *Leymus* *chinensis*, indicating that *C. squarrosa* had higher nutrient acquisition capacity and responded more rapidly to grazing.

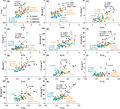

## 1 INTRODUCTION

Grassland ecosystems, covering 40% of the global land area, are one of the most important components of the terrestrial ecosystem, and function as a critical carbon (C) sink (White, Murray, & Rohweder, 2000). Grazing is a traditional grassland management, which has important economic and ecological functions, like providing animal products for human, preventing species invasion in some regions (Kang, Han, Zhang, & Sun, 2007; Kooijman & Smit, 2001). Due to an increase in livestock population, improper grazing management (e.g., overgrazing) gradually turns into a severe threat factor, affecting grassland ecosystem structure, function, and stability (Osem, Perevolotsky, & Kigel, 2002). Grazing has positive or negative effects on soil physicochemical properties (Steffens, Köelbl, Totsche, & Köegel‐Knabner, 2008), soil nutrient cycling (He et al., 2011; Liu et al., 2018; Schuman, Reeder, Manley, Hart, & Manley, 1999), soil respiration (Li et al., 2018; Wang & Fang, 2009), and microbial community composition and structure (Randall et al., 2019), depending on grazing intensity (Han et al., 2008; Schönbach et al., 2011). Moreover, plant functional traits (Li et al., 2015; Zhao, Chen, Han, & Lin, 2009; Zheng et al., 2011), photosynthetic rate, water use efficiency (Chen, Bai, Lin, Liang, & Han, 2005), species composition, community structure, and productivity (Kooijman & Smit, 2001; Osem et al., 2002) are altered with grazing intensity. The nutrient cycling of the soil–plant system plays a critical role in regulating these ecological processes (Dubeux, Sollenberger, Mathews, Scholberg, & Santos, 2007; Schrama et al., 2013).

Soil nutrient availability is important to plant growth and also regarded as a driving force in the alteration of species composition, community structure and function, and even community succession (Koerselman & Meuleman, 1996). However, there are conflicting results on the effect of grazing intensity on soil C, N, and P cyclings in grassland ecosystems. For example, many studies have supported that soil C, N, and P concentrations decreased with grazing intensity, reducing soil nutrient cycling (Han et al., 2008; He et al., 2011; Oliveira Filho et al., 2019). Other studies have shown that soil C, N, and P concentrations increased with grazing intensity, accelerating soil nutrient cycling (Liu et al., 2018; Liu, Zhang, Chang, Kan, & Lin, 2012; Reeder & Schuman, 2002; Reeder, Schuman, Morgan, & Lecain, 2004), while some have suggested that soil nutrient concentrations had no significant response with grazing intensity (Cui et al., 2005; Milchunas & Lauenroth, 1993). Compared with C, N, and P cyclings, there is poorly understanding of microelement dynamic in soil with grazing intensity, which affects plant community structure and function. Moreover, previous studies about soil nutrient cycling under different grazing intensities are examined by the spatial sequence instead of temporal sequence, which is lack of accurately control to background information (e.g., soil texture, vegetation heterogeneity, and grazing intensity) (Cui et al., 2005; Han et al., 2008; Steffens et al., 2008).

The impact of grazing on plant growth is not only limited by the changes in soil nutrient cycling but also extends to direct physical disturbance, like trampling and foraging (Liang, Gornish, Mariotte, Chen, & Liang, 2019; Ritchie, Tilman, & Knops, 1998). Plants must rapidly respond to these disturbances by regulating nutrient strategies, such as C, nitrogen (N), and phosphorus (P) concentrations and their stoichiometry (Liang et al., 2019; Yin et al., 2010). However, plant nutrient concentrations under different grazing intensities remain controversial (He et al. 2019; Han et al., 2008; Ma et al., 2019). For example, some studies have shown plant N and P concentrations increased and plant N/P ratio decreased with grazing intensity (Li et al., 2016), while others reported the opposite result (He et al., 2019). Microelements are essential elements of some functional compounds in plants. For example, Copper (Cu) and iron (Fe) are important components in chlorophyll, some enzymes, and proteins (DalCorso, Manara, Piasentin, & Furini, 2014; Hänsch & Mendel, 2009). Many microelements are also involved in the primary and secondary metabolic processes of plants (Hänsch & Mendel, 2009), especially in N and P metabolisms (DalCorso et al., 2014). Thus, more attention needs to be paid to these microelements, which regulate plant growth and physiological activity (e.g., photosynthesis and respiration) (DalCorso et al., 2014). Understanding the multi‐nutrient dynamics in plants is helpful to elucidate the adaptation of plants to grazing and reveals the underlying mechanisms of changes in community structure and function.

The grassland in Inner Mongolia is an important piece of the Eurasian grassland ecosystem and is representative of grasslands in northern China. (Kang et al., 2007; Li, Jäschke, Guo, & Wesche, 2019). In the past three decades, overgrazing and change in grazing management practices (i.e., seminomadic farming systems to intensified settled livestock farming) have degraded approximately 90% of the grassland (Li et al., 2019). Previous studies have shown the nutrient cycling of the soil–plant system remained uncertain in grassland ecosystems in Inner Mongolia (Cui et al., 2005; Han et al., 2008; He et al., 2011). Moreover, plant species have various nutrient strategies to grazing due to selective foraging (Ritchie et al., 1998). Unfortunately, the responses of multi‐nutrients in different plant species to grazing are poorly understood.

This study aims to clarify the changes in multiple elements of soil and plants with grazing intensity in grassland ecosystems in Inner Mongolia. A grazing experiment with four grazing intensities was conducted in 2015—2016 and the concentrations of eight nutrient elements (C, N, P, Mg, Cu, Fe, Mn, and Zn) in soil and plants were measured and analyzed. We addressed the following questions: (a) Whether macroelements and microelements in the soil–plant system have the same dynamic with grazing intensity? (b) Is the response pattern of these multiple elements consistent across different plant species?

## 2 MATERIALS AND METHODS

### 2.1 Study sites

The study site (44°40.66′N, 116°28.32′E, a.s.l. 1,100 m) was located in a typical grassland in Inner Mongolia, China. The mean annual temperature of the study site is 0.5–1.0°C, with mean monthly temperatures ranging from −19.0°C (January) to 21.4°C (July). The mean annual precipitation is 280.5 mm, approximately 80% of which occurs during the growing season (May to September). The mean annual potential evaporation is about 1,600 to 1,800 mm. According to Chinese soil classification, the soil is chestnut. The dominant species is *Stipa krylovii*, and other species include *Leymus chinensis*, *Cleistogenes squarrosa*, *Carex duriuscula*, *Potentilla tanacetifolia*. *S. krylovii*, *Leymus chinensis*, and *Cleistogenes squarrosa* are perennial grasses and the aboveground biomass of these three species accounts for 70% of the total community aboveground biomass and greatly affects community structure and function. Grazing exclusion occurred from 2007 to 2012 in this region, and the aboveground biomass was harvested at the termination of the growing season once a year.

### 2.2 Experimental design

We selected a flat area with uniform soil and vegetation and established 12 independent grazing plots (each of which was 1.33 hm^2^), arraying four rows and three columns in March of 2012. The grazing experiment ran from June 10th to September 10th of each year (2012—) and the sheep were continuously stocked all day during this period. According to the *calculation of proper carrying capacity of rangeland* (NY/T635‐2002), grazing intensity was achieved by the standard sheep unit (SSU), defined as 2‐year female sheep with 50‐kg. In this study, the sheep were two‐year‐old, 31 kg female and were transformed to SSU. Grazing intensity was calculated by following equation:

$$\text{Grazing}\;\text{intensity}=\frac{W\times N\times D}{W_{\text{SSU}}\times S}$$

where *W* is the weight of experimental sheep (31 kg), *N* is the number of experimental sheep (0, 4, 8, and 16 experimental sheep in one plot, respectively), *D* is the grazing time (91 days), W_SSU_ is the weight of standard sheep (50 kg), and S is the area of grazing plot (1.33 hm^2^). Grazing intensity included CK—ungrazed control (0 SSU day/year hm^2^), LG—light grazing (170 SSU day/year hm^2^), MG—moderate grazing (340 SSU day/year hm^2^), and HG—heavy grazing (680 SSU day/year hm^2^). Four grazing intensities were randomly assigned to the grazing plots with three replicates.

### 2.3 Sample collection and measurements

Soil and plant samples were collected at the time of peak aboveground biomass (August 15th of 2015 and 2016). Five sampling quadrats (1 m × 1 m) were equidistantly set 30 m apart in the diagonal of each grazing plot. In each quadrat, five mature and healthy plant individuals of each species (*S. krylovii*, *L. chinensis*, and *C. squarrosa*) were randomly selected and the aboveground part of each plant individual was clipped. The soil samples were taken from depths of 0–10 cm, 10–20 cm, 20–30 cm in the center of each quadrat by using a 3‐cm‐diameter soil auger after plant sampling. Same species plant samples and soil samples of the same depth from each plot were uniformly mixed. The plant samples were oven‐dried at 65°C for 48 hr, and the soil samples were air‐dried in a laboratory after removing plant roots and stones.

Plant and soil samples were ground with a mill (MM400; Retsch). Total C and N concentrations were measured by a Vario EL (vario EL III CHNOS Elemental Analyzer, Elementar Analysensysteme GmbH). Total P, Mg, Cu, Fe, Zn, and manganese (Mn) concentrations were determined by using ICP‐OES (iCAP 6300 ICP‐OES Spectrometer, Thermo Scientific). Soil available P and N concentrations were measured by an ultraviolet spectrophotometer (UV‐2550, UV‐Visible Spectrophotometer, Shimadzu) and alkaline hydrolysis diffusion, respectively. In this study, soil available N mainly includes ammonia nitrogen, nitrate nitrogen, amino acids, amides, and easily hydrolyzed proteins.

### 2.4 Data analysis

Relative increase ratio indicates the change in nutrient concentrations of plants under different grazing intensities, calculated by the following equation:

$$\text{Relative}\;\text{increase}\;\text{ratio}=\frac{C_{\text{GI}}‐C_{\text{CK}}}{C_{\text{CK}}}$$

where C_GI_ indicates the mean of an element concentration of plant under a grazing intensity and C_CK_ indicates the mean of this element concentration under ungrazed control.

Because of short‐term grazing (2012—2015), we paid more attention to the effect of grazing intensity on the nutrient concentrations of soil and plants compared with time effect. Levene's test was used to examine the homogeneity of variances for all data. One‐way ANOVA with liner polynomial contrast was selected to compare the differences in nutrient concentrations of soil and plants among different grazing intensities. One‐variable linear regression was selected to quantify the relationships between plant N and P and other nutrient elements (i.e., Mg, Cu, Fe, Mn, and Zn). Data were indicated by mean ± 1*SE* (*n* = 3), and the significance level of data analysis was 0.05. All data analyses were conducted by SPSS 21.0 (SPSS Inc.).

## 3 RESULT

### 3.1 Response of soil nutrients to different grazing intensities

**FIGURE 1:**
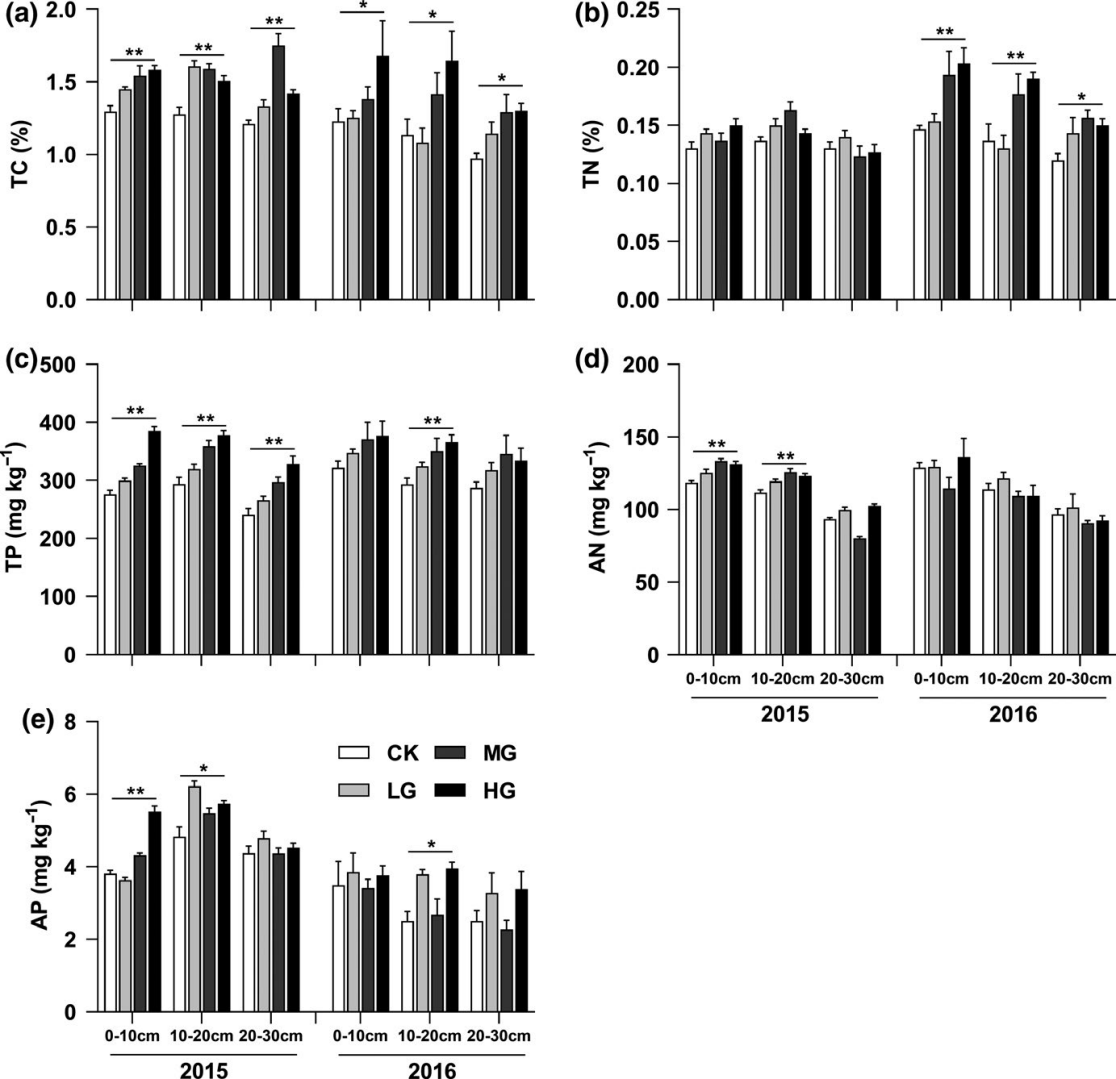
Vertical distribution of soil TC, TN, TP, AN, and AP concentrations in the grassland under different grazing intensities. AN, available nitrogen; AP, available phosphorus; TC, total carbon; TN, total nitrogen; TP, total phosphorus; *,** indicated soil macroelement concentrations had linear trends with grazing intensity at the 0.05 and 0.01 level, respectively

Macroelements and microelements in soil had inconsistent responses to grazing intensity. Specifically, the means of soil total C, total N, total P, available N, and available P concentrations significantly increased with grazing intensity (Figure 1; *p* < .05), especially at the depth of 0–10 cm. However, the means of soil Mg, Cu, Fe, Mn, and Zn concentrations had no significant response with grazing intensity (Figure S1).

### 3.2 Response of plant nutrients to different grazing intensities

**FIGURE 2:**
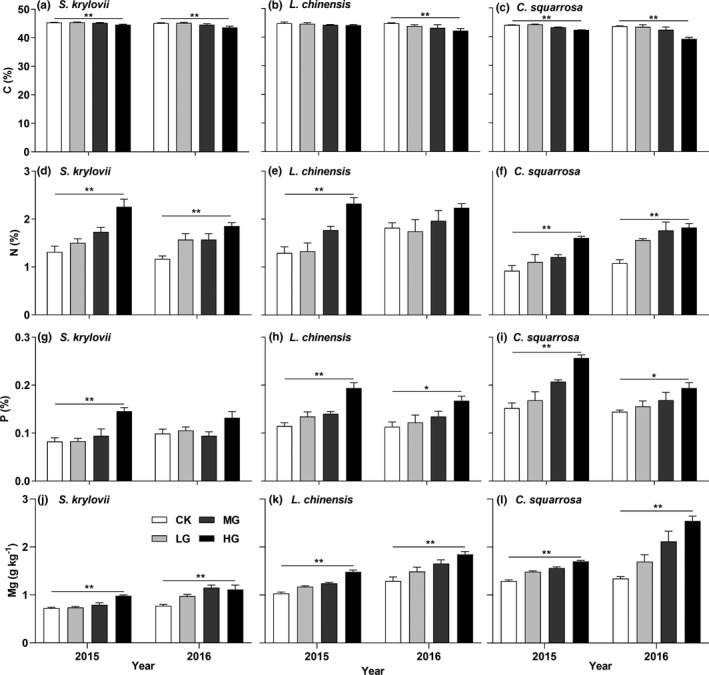
Macroelement concentrations of the three species in the grassland under different grazing intensities. *,** indicated plant macroelement concentrations had linear trends with grazing intensity at the 0.05 and 0.01 level, respectively

Macroelements in plants showed different responses to grazing intensity. The mean of plant C concentration remarkably decreased with grazing intensity (Figure 2a–c; *p* < .05). In contrast, the means of plant N, P, and Mg concentrations significantly increased with grazing intensity (Figure 2d–l; *p* < .05).

**FIGURE 3:**
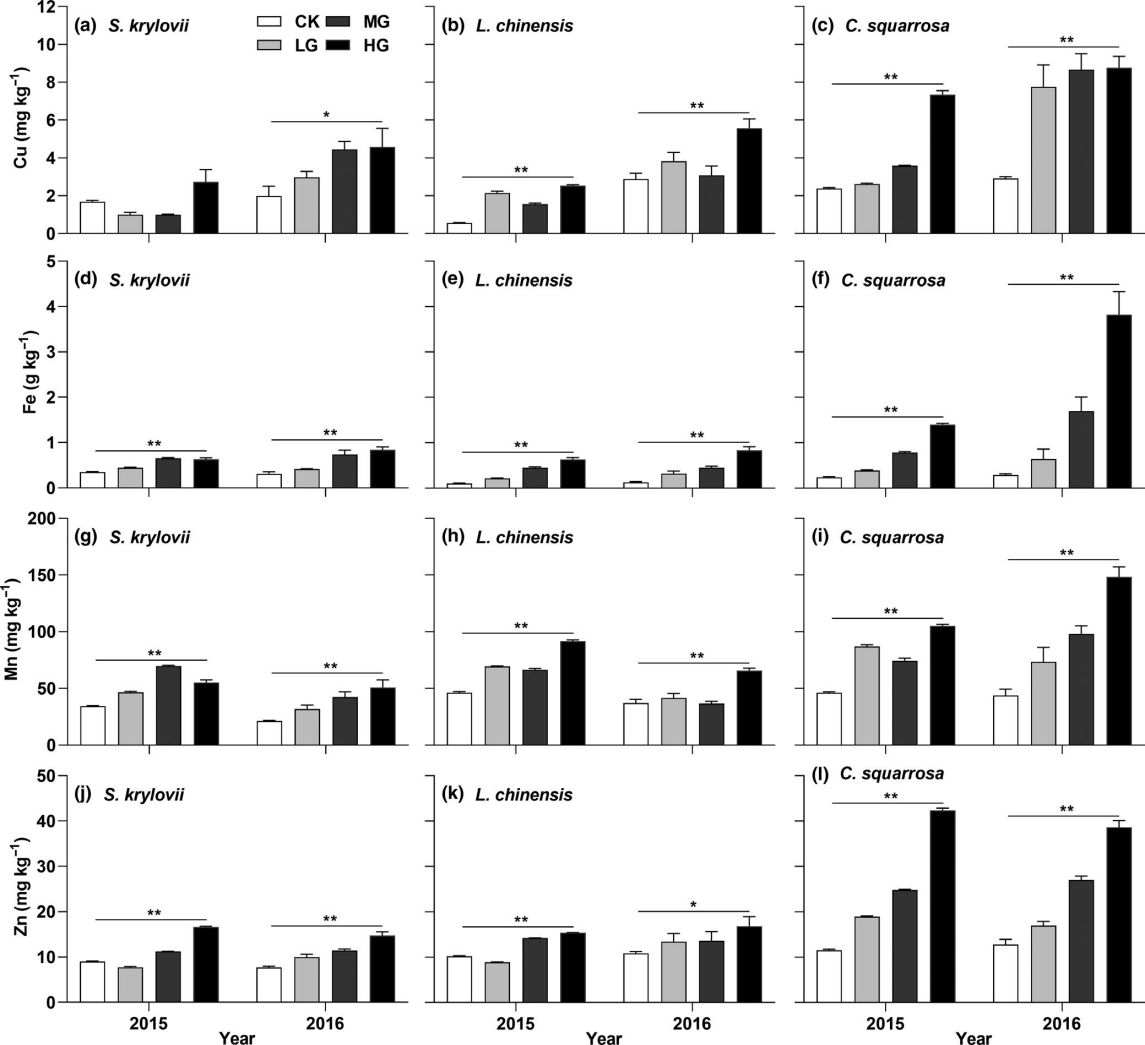
Microelement concentrations of the three species in the grassland under different grazing intensities. *,** indicated plant microelement concentrations had linear trends with grazing intensity at the 0.05 and 0.01 level, respectively

Microelements in plants had the same response to grazing intensity. The means of plant Cu, Fe, Mn, and Zn concentrations linearly increased with grazing intensity (Figure 3; *p* < .05). Compared with C, N, P, and Mg, the means of plant Cu, Fe, Mn, and Zn concentrations exhibited higher relative increase ratios under different grazing intensities (Table 1).

**TABLE 1:** Relative increase ratio of nutrient elements in the three species under different grazing intensities

Species	Element	2015			2016		
		LG	MG	HG	LG	MG	HG
*S. krylovii*	C	0.1%	−0.5%	−1.7%	0.2%	−1.3%	−3.5%
*L. chinensis*		−0.4%	−1.4%	−1.5%	−2.1%	−3.5%	−5.8%
*C. squarrosa*		0.4%	−1.9%	−3.9%	−0.7%	−3.0%	−10.2%
*S. krylovii*	N	14.2%	31.6%	71.4%	34.2%	34.5%	58.4%
*L. chinensis*		2.6%	36.8%	79.2%	−4.0%	8.1%	22.9%
*C. squarrosa*		20.4%	31.0%	74.5%	44.4%	63.2%	68.8%
*S. krylovii*	P	0.4%	14.1%	76.2%	6.7%	−4.7%	33.3%
*L. chinensis*		17.1%	22.0%	68.7%	7.9%	18.8%	47.9%
*C. squarrosa*		10.7%	36.1%	68.5%	7.6%	16.8%	34.1%
*S. krylovii*	Mg	2.3%	9.7%	36.4%	25.9%	49.1%	44.4%
*L. chinensis*		13.5%	20.3%	43.9%	15.2%	28.1%	43.0%
*C. squarrosa*		15.2%	21.2%	31.8%	26.3%	57.6%	89.6%
*S. krylovii*	Cu	−40.8%	−40.6%	62.6%	49.9%	123.9%	130.6%
*L. chinensis*		282.7%	176.8%	350.6%	32.4%	6.8%	93.0%
*C. squarrosa*		9.5%	50.3%	208.0%	165.7%	197.1%	200.3%
*S. krylovii*	Fe	28.8%	87.5%	81.7%	35.5%	138.7%	172.0%
*L. chinensis*		100.0%	325.0%	490.6%	143.6%	246.2%	543.6%
*C. squarrosa*		64.8%	233.8%	490.1%	120.7%	485.1%	1,218.4%
*S. krylovii*	Mn	35.4%	103.2%	61.1%	49.6%	99.9%	139.2%
*L. chinensis*		50.8%	44.3%	98.9%	11.9%	−1.7%	76.3%
*C. squarrosa*		89.3%	61.5%	128.1%	67.4%	123.2%	237.5%
*S. krylovii*	Zn	−14.4%	24.3%	84.1%	30.8%	49.0%	91.9%
*L. chinensis*		−12.9%	39.2%	50.6%	24.6%	26.4%	56.1%
*C. squarrosa*		64.7%	115.9%	268.4%	32.6%	111.0%	201.9%

**FIGURE 4:**
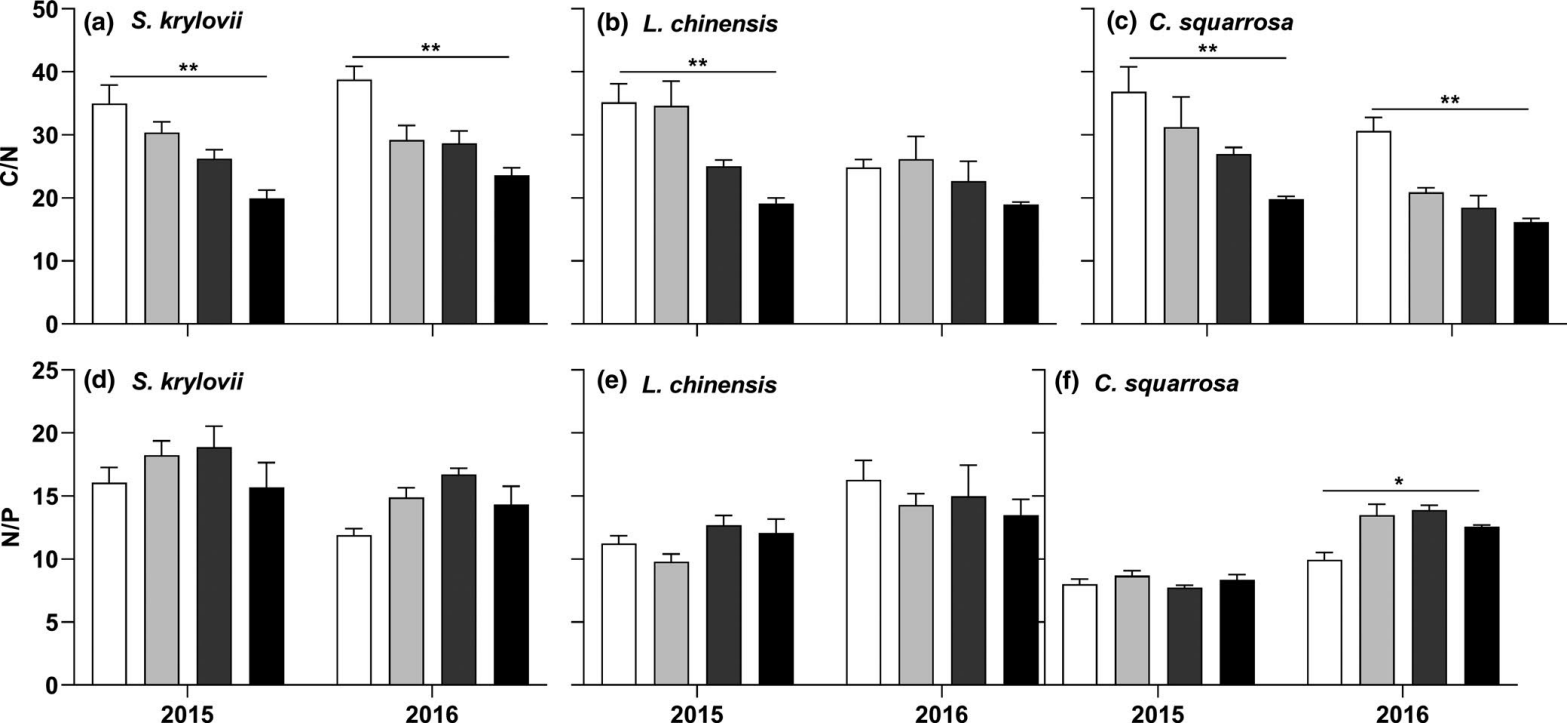
C/N ratio and N/P ratio of the three species in the grassland under different grazing intensities. *,** indicated plant stoichiometry had linear trends with grazing intensity at the 0.05 and 0.01 level, respectively

Plant C/N ratio in the three species remarkably decreased with grazing intensity (Figure 4a–c; *p* < .05), while plant N/P ratio had no consistent response (Figure 4d–f).

### 3.3 Coupling effects of nutrient elements under different grazing intensities

**FIGURE 5:**
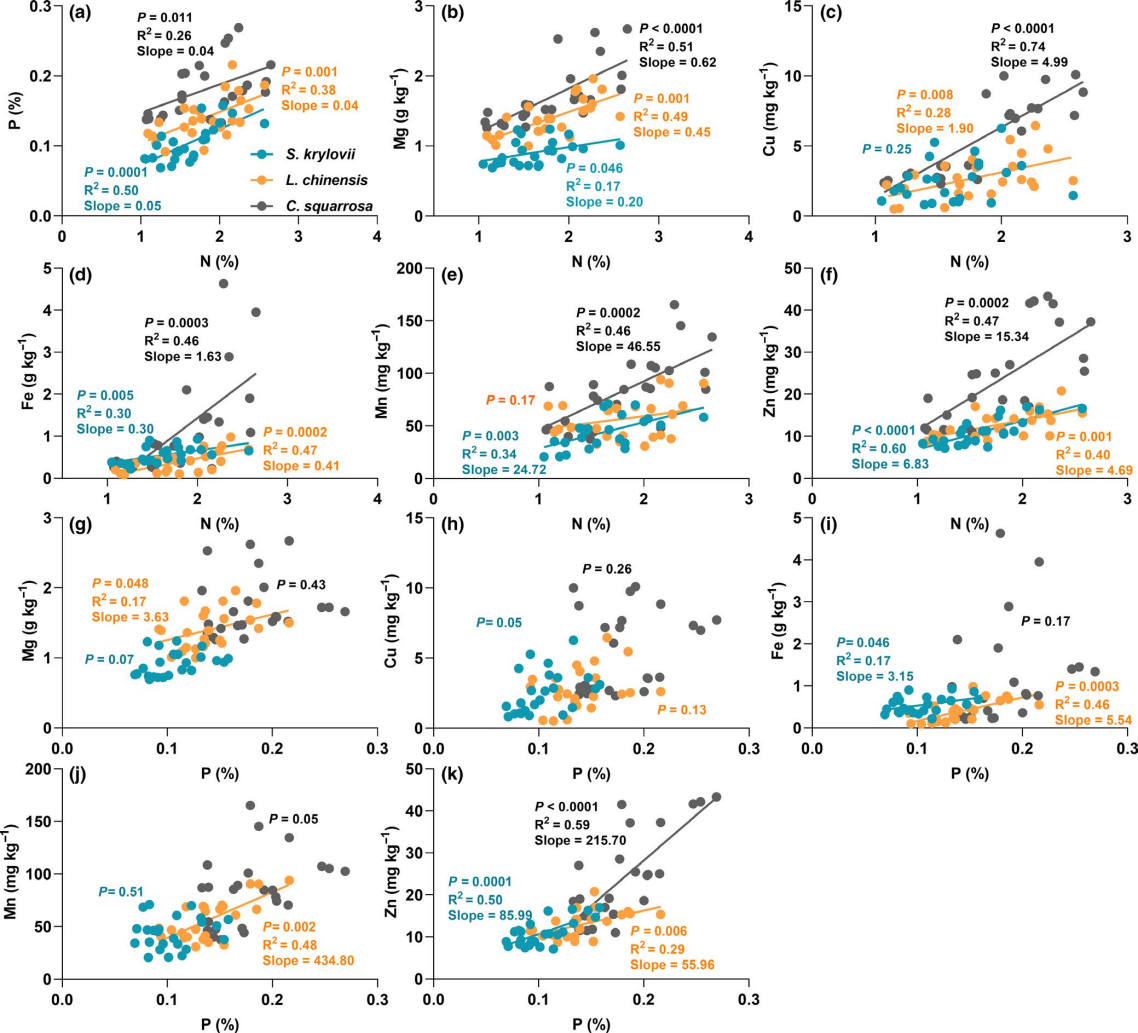
Relationships between plant N and P concentrations and other element concentrations under different grazing intensities

Among the nutrient elements, N exhibited significantly positive correlations with P, Mg, Cu, Fe, and Zn (Figure 5a–f; *p* < .05) in the three species with grazing intensity, except for N and Cu in *S. krylovii* and N and Mn in *L. chinensis*. P was positively related to Zn (Figure 5k; *p < *.05), while no significant relationship between P and Mg, Cu, Fe, and Mn was observed (Figure 5g–j). Nutrient elements in the three species responded differently to grazing intensity. Compared with *S. krylovii* and *L. chinensis*, the nutrient concentrations in *C. squarrosa* were more sensitive to grazing intensity (Table 1). For example, the relative increase ratio of Mn in *C. squarrosa* was 128.1% and 237.5% in 2015 and 2016 under heavy grazing, respectively, which was higher than that in *S. krylovii* (61.1% and 139.2%) and *L. chinensis* (98.9% and 76.3%) (Table 1). More importantly, the slopes of nutrient linear regression in *C. squarrosa* were higher than those in *S. krylovii* and *L. chinensis* (Figure 5).

## 4 DISCUSSION

This study supports the hypothesis that the nutrient concentrations of the soil–plant system increase with grazing intensity in grassland ecosystems. The nutrient dynamics of macroelements and microelements in soil and plants displayed opposite responses to grazing intensity. In the soil system, the nutrient dynamics of soil C, N, and P concentrations were more sensitive than microelements. In the plant system, Cu, Fe, Mn, and Zn concentrations exhibited significant increases. Moreover, the nutrient concentrations of different species had asymmetric responses to grazing intensity. *C. squarrosa* revealed higher slopes of nutrient linear regression than *S. krylovii* and *L. chinensis*, indicating *C. squarrosa* had higher nutrient acquisition capacity and more intensive response to heavy grazing.

### 4.1 Effect of grazing on plant C, N, and P

Plant C concentration significantly reduced with grazing intensity in this study, while previous studies found plant C concentration had no significant response to grazing intensity (Ding, Gong, Wang, Wu, & Liu, 2012; Liang et al., 2019). Grazing intensity is a critical factor to regulate plant C dynamic (He et al., 2019). Compared with Liang et al. (2019)'s study, higher grazing intensity had stronger damage to plant stems and leaves in this study, which could decrease accumulation of organic matter. The reconstruction of plant stem and leaf foraged by livestock also consumed much carbohydrate, thus contributing to the decrease in plant C concentration. Plant N and P concentrations increased with grazing intensity (Figure 3a–i), which aligned with findings from previous studies (Li et al., 2016; Zheng, Ren, Li, & Lan, 2012). Because mature and senescent plant tissue was foraged and plants need amounts of N and P to restructure stem and leaf (Ma et al., 2019). The new growth, comprised of young leaves and tillers, contained higher N and P concentrations, particularly in heavy grazing. Moreover, the increase in soil nutrient concentrations (Figure 1) might indirectly accelerate plant growth, which also increased plant N and P concentrations.

We found that plant C/N ratio significantly decreased with grazing intensity (Figure 4a–c; *p* < .05), which was consistent with previous studies (Bai et al., 2012; Zheng et al., 2012). It confirmed that grazing accelerated N cycling and plant C/N ratio could indicate plant nutrient strategies to grazing disturbance. However, plant N/P ratio had no consistent response with grazing intensity (Figure 4d–f). This result indicated plant growth suffered from short‐term grazing was not limited by N or P, compared with long‐term grazing (He et al., 2019; Yin et al., 2010). The acceleration of soil nutrient cycling might provide enough N and P for plant growth. However, He et al. (2019) reported that plant N/P ratio decreased with grazing intensity. These inconsistent results showed that plant N/P ratio might be an uncertain indicator of plant growth limitations in grazing grasslands, depending on grazing intensity and time.

### 4.2 Effect of grazing on plant Mg, Cu, Fe, Mn, and Zn

Plant Mg, Cu, Fe, Mn, and Zn concentrations significantly increased with grazing intensity (Figure 3; *p* < .05). These nutrient elements are essential components of some functional material, like chlorophyll, auxin, and various enzymes, which involved in vital important physiological processes (DalCorso et al., 2014; Hänsch & Mendel, 2009). Plant increased chlorophyll concentration, enzyme activity, and photosynthetic rate during the reconstruction of plant stem and leaf, particularly in heavy grazing. Some studies reported that grazing resulted in warming and drying effects on grassland soil (Yan et al., 2018), which might cause plant growth to be stressed by drought. Some microelements (e.g., Cu and Zn) could increase plant drought resistance (DalCorso et al., 2014; Maschner, 1995). Moreover, some microelements were also involved in N and P metabolism (Hänsch & Mendel, 2009), which indirectly increased microelement concentrations with grazing intensity.

We found that microelements had higher relative increase ratios (Table 1), indicating that microelements had more sensitive responses to grazing intensity than C, N, and P. Thus, microelements could play important roles in plant adaptation to grazing. In the future, plant microelements stoichiometry could provide a new tool to study plant adaptation in grassland ecosystems.

### 4.3 Effect of grazing on plant species

Plants had various nutrient strategies to grazing in grassland ecosystems (Liang et al., 2019). Previous studies reported that long‐term heavy grazing decreased the dominance of *L. chinensis* and *S. grandis*, resulting in a surplus of community space and resources. *C. squarrosa* used these resources to reproduce rapidly, thereby degrading the grasslands (Wang, Liang, Liu, & Hao, 2000). We found that the slopes of nutrient linear regression in *C. squarrosa* were higher than that in *S. krylovii* and *L. chinensis* (Figure 5). Higher nutrient concentrations in *C. squarrosa* might have a higher photosynthetic rate, protein and nucleic acid synthesis capacity, nutrient use efficiency, and drought resistance, which renders them more tolerant to heavy grazing. This result indirectly suggested that *C. squarrosa* had a higher capacity of nutrient acquisition under heavy grazing. Nutrient dynamics of different species might explain the changes in species composition and community structure during grassland degradation from the perspective of plant nutrient utilization.

### 4.4 Effect of grazing on soil nutrients

In soil, grazing had more notable effects on macroelement concentrations than those of microelements. Short‐term heavy grazing could accelerate the decomposition of plant litter, feces, and urine via livestock trample, which directly increased the input of organic matter and stimulated the mineralization of C, N, and P (Liu et al., 2018; Steffens et al., 2008). In addition, Hamilton and Frank (2001) reported that grazing increased the root exudation of carbon and increased the number of soil microbes. These reasons resulted that soil total C, total N, total P, available N, and available P concentrations increased with grazing intensity (Figure 1), supporting part of studies (Liu et al., 2018; Reeder & Schuman, 2002; Reeder et al., 2004). In contrast, soil microelement concentrations had no significant response to grazing intensity (Figure S1), further showing that soil macroelements had sensitive indicators for grazing. This result supported previous studies (Mathews, Sollenberger, Nair, & Staples, 1994).

Interestingly, He et al. (2011) and Steffens et al. (2008) found soil nutrients decreased with grazing intensity. In this study, 6‐year grazing exclusion before the experiment provided enough organic matter (e.g., plant litter) for soil nutrient cycling. In addition, grassland ecosystems possess a certain elasticity and threshold to grazing. Short‐term heavy grazing had positive effects on soil nutrients (Liu et al., 2012), while extended grazing time could have negative effects on the nutrient balance of the soil–plant system (He et al., 2011; McSherry & Ritchie, 2013; Steffens et al., 2008). Thus, compared with plant nutrients, the impacts of grazing intensity on soil nutrients were complex, depending on land use pattern, community type, and grazing intensity and time (Reeder & Schuman, 2002). In the future, the effect of grazing on soil nutrients should be long‐term and continuous observation.

## 5 CONCLUSIONS

This study broadens the knowledge of the multiple element dynamics in the soil–plant system under different grazing intensities in grassland ecosystems, especially among microelements and highlights the importance of microelements in plant adaptation to grazing. Our results demonstrated that short‐term heavy grazing comprehensively increased the concentrations of both macroelements and microelements in the soil–plant system, accelerating nutrient cycling of grassland ecosystems. Plant nutrients had more sensitive and rapid responses with grazing intensity than soil. More importantly, macroelement concentrations in soil were more sensitive than microelements with grazing intensity, while the opposite is true in plants, with microelements displaying a higher sensitivity. Microelements and their stoichiometry could provide a new insight to study plant adaptation to grazing in grassland ecosystems. Compared with *L. chinensis* and *S. krylovii*, *C. squarrosa* exhibited higher slopes of nutrient linear regression, suggesting more tolerance and adaptability under heavy grazing via increasing nutrient acquisition.

## CONFLICT OF INTEREST

No conflict of interest.

## AUTHOR CONTRIBUTION

**Dongjie Hou:** Conceptualization (supporting); Data curation (lead); Formal analysis (lead); Funding acquisition (supporting); Investigation (lead); Methodology (equal); Project administration (supporting); Resources (supporting); Software (lead); Supervision (supporting); Validation (supporting); Visualization (lead); Writing‐original draft (lead); Writing‐review & editing (lead). **Ke Guo:** Conceptualization (equal); Data curation (supporting); Formal analysis (equal); Funding acquisition (equal); Investigation (supporting); Methodology (equal); Project administration (lead); Resources (lead); Software (supporting); Supervision (lead); Validation (equal); Visualization (supporting); Writing‐original draft (equal); Writing‐review & editing (equal). **Changcheng Liu:** Conceptualization (lead); Data curation (supporting); Formal analysis (equal); Funding acquisition (equal); Investigation (supporting); Methodology (equal); Project administration (equal); Resources (equal); Software (supporting); Supervision (equal); Validation (equal); Visualization (supporting); Writing‐original draft (equal); Writing‐review & editing (equal).

## Supporting information

Fig S1

## Data Availability

Data associated with this paper has been uploaded in Dryad: https://doi.org/10.5061/dryad.37pvmcvgz.
