# Supplementary figures and images for: Asymmetric effects of grazing intensity on macroelements and microelements in grassland soil and plants in Inner Mongolia Grazing alters nutrient dynamics of grasslands
Source: Ecol Evol. 2020 Jul 18;10(16):8916–26. doi: 10.1002/ece3.6591 (PMC7452780; doi:10.1002/ece3.6591)

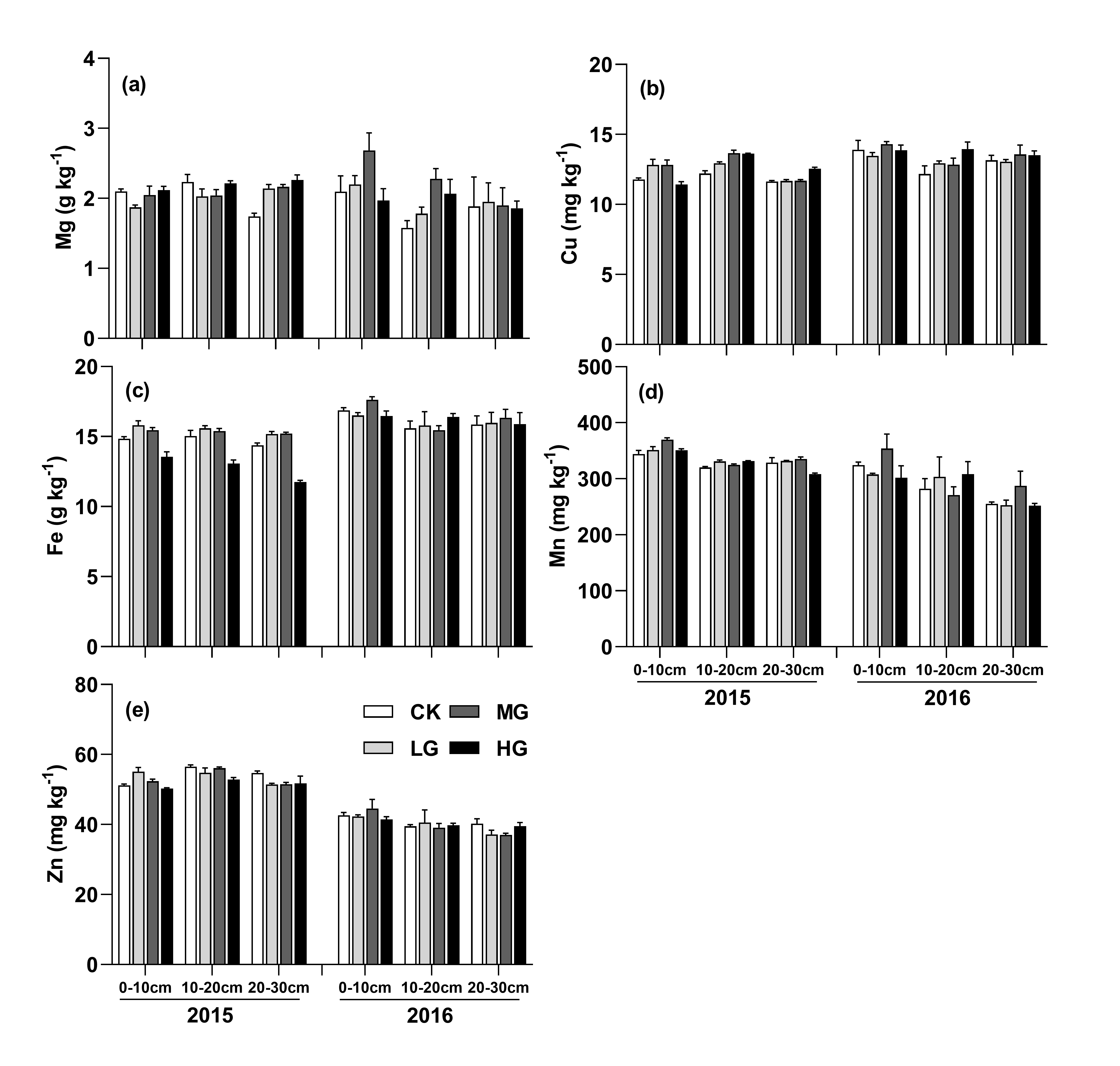

Supplement: Supplementary file 1 — Fig S1 [file ECE3-10-8916-s001.tif]
